# Ruptured Vertebral-Anterior Spinal Artery Junction Aneurysm Presenting With Diffuse Subarachnoid Hemorrhage: Diagnostic Limitations of CT Angiography (CTA) and the Role of Digital Subtraction Angiography (DSA) in Management-Related Decision-Making

**DOI:** 10.7759/cureus.99170

**Published:** 2025-12-13

**Authors:** Jedediah Bondy, Muhammad Altaf, Yaqub Murad, Nirav Patel, Sara K Roshan

**Affiliations:** 1 Radiology, Lake Erie College of Osteopathic Medicine, Erie, USA; 2 Radiology, ChristianaCare, Newark, USA; 3 Neuroradiology, ChristianaCare, Newark, USA

**Keywords:** 3d rotational angiography, anterior spinal artery aneurysm, complicated subarachnoid hemorrhage, digital subtraction angiography(dsa), neurocritical care

## Abstract

Subarachnoid hemorrhage (SAH) is most commonly caused by rupture of aneurysms arising from the anterior circulation. Aneurysms involving the anterior spinal artery (ASA) are exceptionally rare and pose unique diagnostic and therapeutic challenges due to their small size, deep midline location, and critical role in spinal cord perfusion. We report the case of a 67-year-old female who presented with a sudden thunderclap headache and diffuse SAH centered in the prepontine cistern with intraventricular extension. CT angiography (CTA) demonstrated a subtle vascular irregularity near the left vertebral-ASA junction, but could not definitively characterize the lesion due to posterior fossa artifact. Digital subtraction angiography (DSA) confirmed a 1.5-2 mm ruptured aneurysm arising from the ASA at its junction with the dominant left vertebral artery and demonstrated functional bilateral ASA collateral supply. Given the high risk of spinal cord ischemia with endovascular treatment, conservative management was selected. The patient's condition stabilized under neurocritical care, and she was discharged to rehabilitation with improved neurological function. This report highlights the limitations of CTA for small posterior circulation aneurysms and underscores the essential role of DSA and ASA collateral assessment in guiding individualized management.

## Introduction

Subarachnoid hemorrhage (SAH) is a life-threatening neurological emergency, typically presenting with sudden, severe headache, nausea, vomiting, and altered consciousness [1]. While traumatic SAH is most common overall, spontaneous non-traumatic SAH is most frequently caused by rupture of an intracranial aneurysm [2]. Approximately 80-85% of ruptured aneurysms arise from the anterior circulation, while posterior circulation aneurysms are far less common but carry higher morbidity due to their proximity to the brainstem and vital perforating vessels [3].

The anterior spinal artery (ASA) arises from small branches of the intradural vertebral arteries and supplies the lower medulla and anterior two-thirds of the spinal cord [4]. True ASA aneurysms are exceedingly rare, accounting for well under 1% of all intracranial aneurysms [5,6]. Their diagnosis is technically challenging due to their very small caliber, deep midline location, complex vertebrobasilar anatomy, and frequent obscuration by skull-base artifact on noninvasive imaging [6,7,8,9]. As a result, CT angiography (CTA) may raise suspicion but often cannot reliably characterize ASA aneurysms, necessitating digital subtraction angiography (DSA) for definitive diagnosis [10,11]. The objective of this case report is to demonstrate the diagnostic limitations of CTA in detecting small posterior circulation aneurysms and to emphasize the critical role of DSA and ASA collateral assessment in informing safe, individualized management.

## Case presentation

Initial presentation

A 67-year-old female with a medical history of hypertension and migraine presented to the emergency department after experiencing a sudden-onset thunderclap headache accompanied by repeated vomiting while exercising. She denied trauma, seizure activity, anticoagulant use, fever, or recent infection. On examination, she was drowsy but responsive and exhibited expressive aphasia, dysarthria, and symmetric 4/5 strength in all extremities. Her blood pressure was 162/81 mmHg. Shortly after presentation, her mental status deteriorated, and she required endotracheal intubation for airway protection.

Initial imaging and neurocritical care

**Figure 1:**
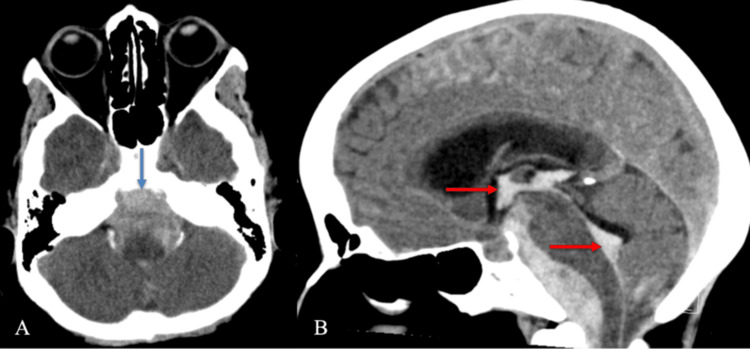
Non-contrast CT head demonstrating diffuse SAH with intraventricular extension (A) Axial non-contrast CT head demonstrates diffuse SAH centered in the prepontine cistern (blue arrow), extending into the basal cisterns and perimesencephalic spaces. (B) ﻿﻿﻿﻿Sagittal non-contrast CT head from the same exam shows intraventricular extension of hemorrhage into the third and fourth ventricles (red arrows), consistent with Modified Fisher grade 4 SAH. Mild ventricular enlargement is present, suggesting early hydrocephalus CT: computed tomography; SAH: subarachnoid hemorrhage

**Figure 2:**
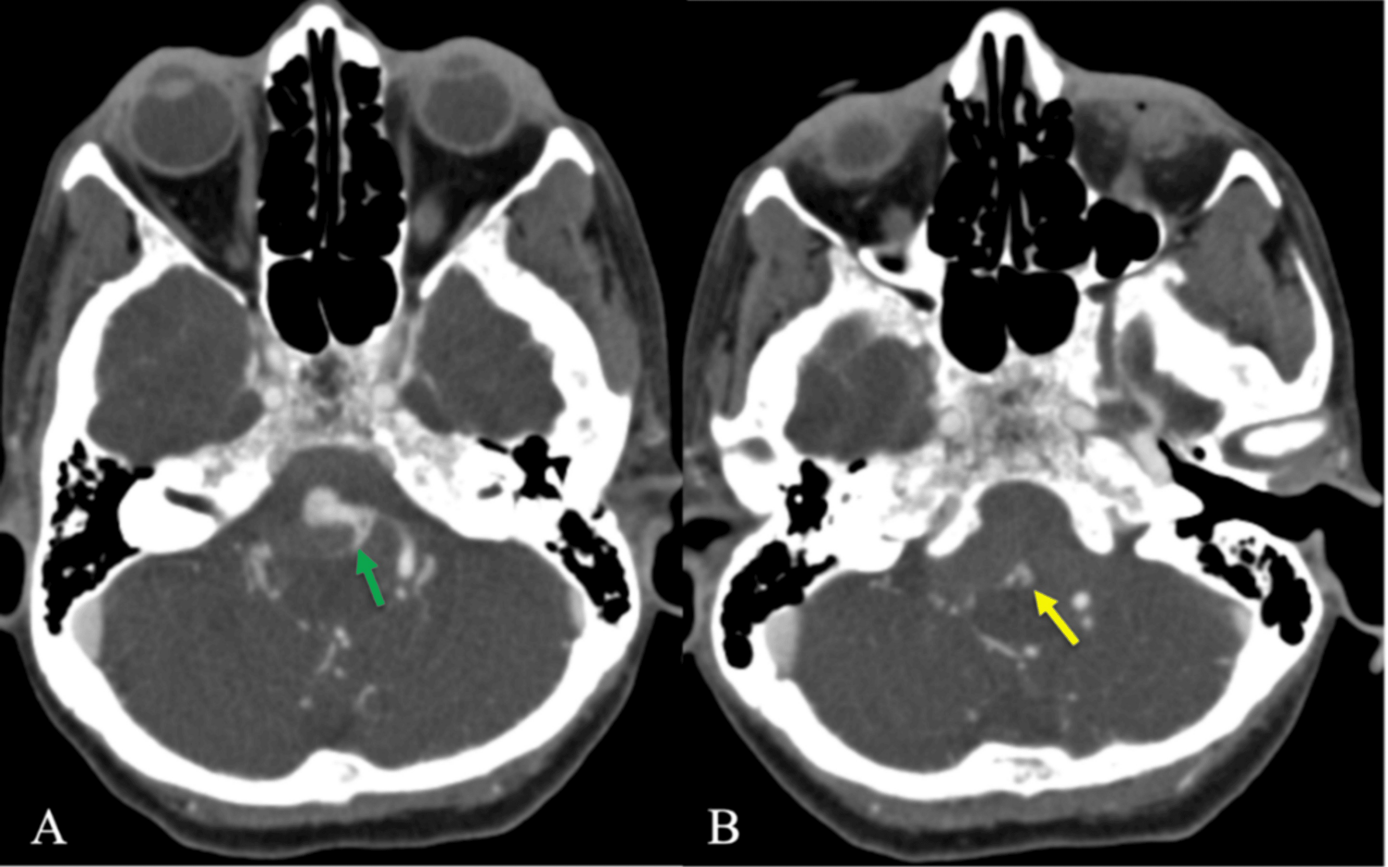
CTA demonstrating left vertebral-anterior spinal artery junction aneurysm (A) ﻿﻿﻿﻿Axial CTA head demonstrates the origin of the anterior spinal artery arising from the distal left V4 segment of the vertebral artery (green arrow). (B) ﻿﻿﻿﻿Axial CTA head at a slightly inferior level shows a small, irregular outpouching arising from the anterior spinal artery, consistent with a 2 mm saccular aneurysm (yellow arrow) CTA: computed tomography angiography

Non-contrast CT of the head demonstrated diffuse SAH centered within the basal and prepontine cisterns, with intraventricular extension into the third and fourth ventricles and early hydrocephalus, consistent with high-grade aneurysmal SAH (Figure 1) [7,8]. No midline shift or transtentorial herniation was present. CTA of the head and neck demonstrated a subtle focal vascular irregularity along the distal intradural left vertebral artery near the expected origin of the ASA (Figure 2). Due to posterior fossa beam-hardening artifact and overlapping vertebrobasilar vasculature, a definitive aneurysm could not be confidently characterized [9,10].

The patient was admitted to the neurocritical care unit and treated with nimodipine for vasospasm prophylaxis, levetiracetam for seizure prophylaxis, and nicardipine infusion for blood pressure control. An external ventricular drain (EVD) was placed for the management of obstructive hydrocephalus with effective cerebrospinal fluid diversion [7].

Definitive angiographic diagnosis

**Figure 3:**
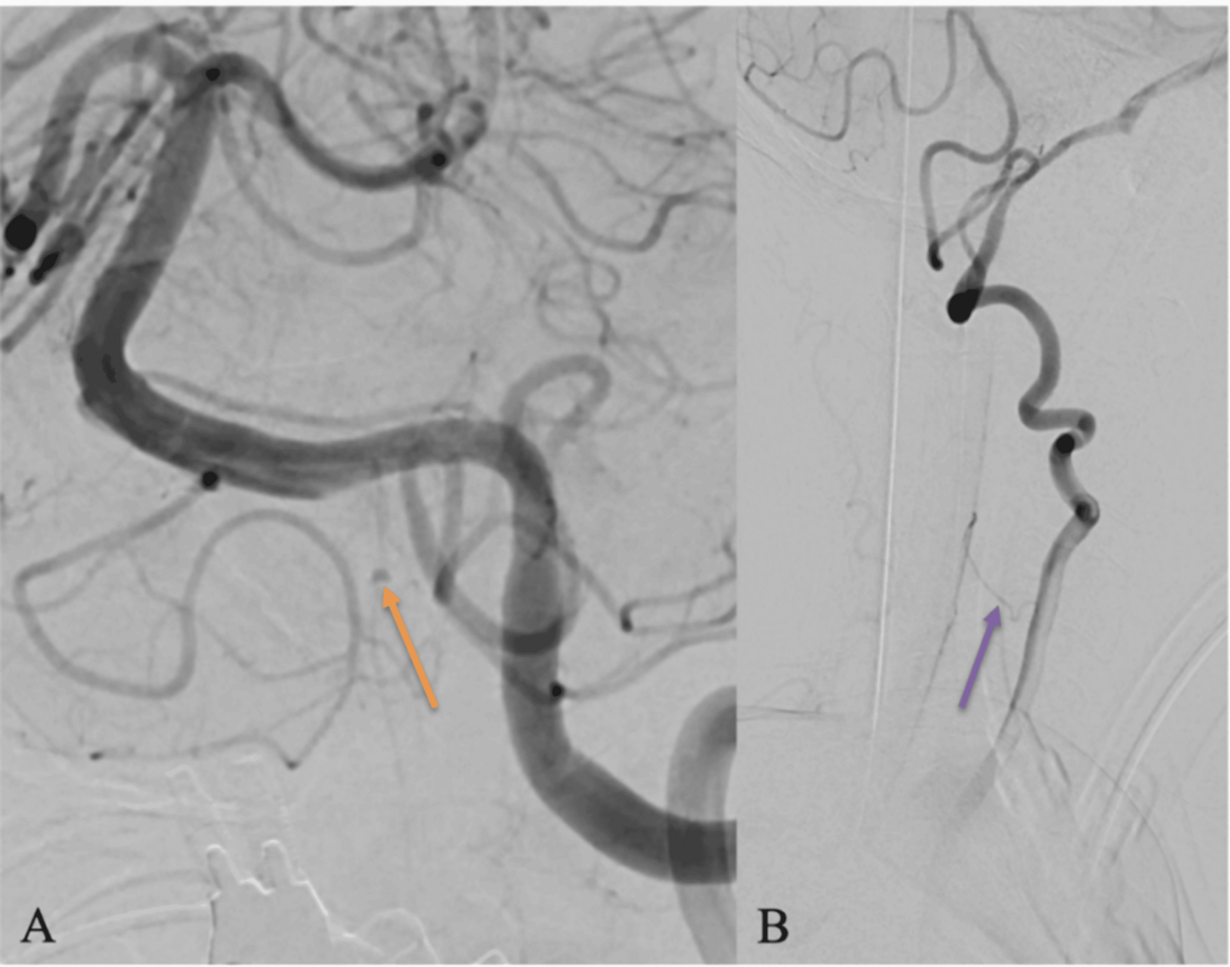
DSA confirming left vertebral-anterior spinal artery aneurysm and collateral flow (A) Magnified lateral projection demonstrates a 1.5 mm irregular aneurysm (orange arrow) arising from the anterior spinal artery at its junction with the left vertebral artery, consistent with a ruptured ASA aneurysm. (B) Oblique projection demonstrates a collateral vessel (purple arrow) from the contralateral vertebral artery supplying the ASA, confirming bilateral perfusion and explaining the decision to avoid endovascular occlusion DSA: digital subtraction angiography

**Figure 4:**
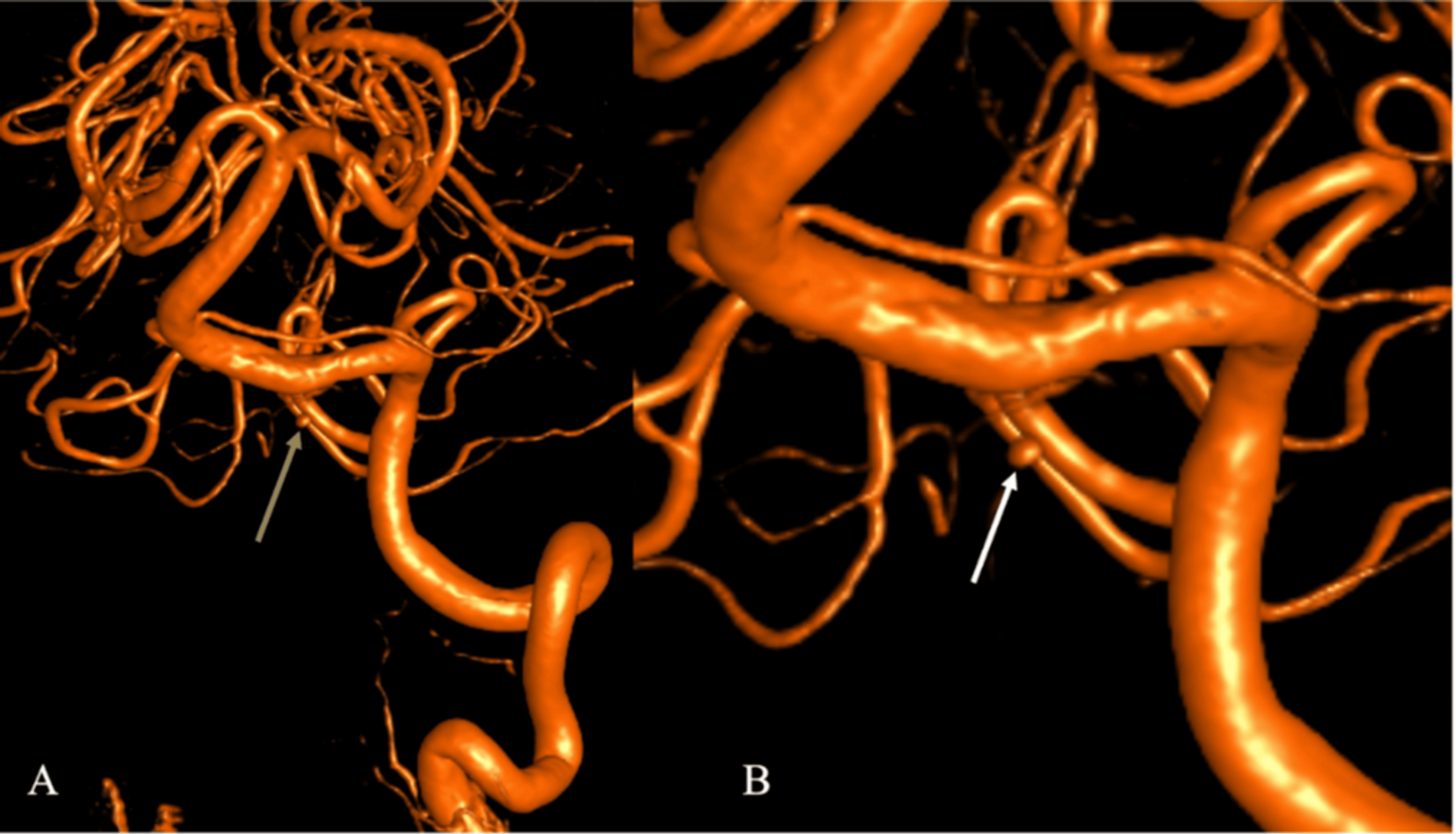
Three-dimensional rotational DSA (3D-DSA) of the left vertebral-anterior spinal artery junction aneurysm (A) Three-dimensional reconstruction of the left vertebral artery demonstrates a 1.5-2 mm saccular aneurysm (tan arrow) arising from the anterior spinal artery at its vertebral junction. (B) Magnified view provides detailed delineation of the aneurysm neck and morphology (white arrow), confirming its midline projection and proximity to the ASA origin DSA: digital subtraction angiography; ASA: anterior spinal artery

DSA performed the following morning confirmed a 1.5-2 mm saccular aneurysm arising directly from the ASA at its junction with the dominant left vertebral artery (Figure 3A). The contralateral vertebral artery was structurally normal but demonstrated functional collateral perfusion of the ASA on oblique projections, confirming bilateral supply (Figure 3B). Three-dimensional rotational angiography provided additional delineation of the aneurysm neck and its direct continuity with the ASA origin (Figure 4) [10].

Management and clinical course

Because the aneurysm arose directly from the ASA trunk and the vessel provides dominant perfusion to the anterior spinal cord, endovascular occlusion or stent-assisted intervention carried a substantial risk of catastrophic spinal cord ischemia [8,12]. Given the patient’s clinical stabilization under neurocritical care and the prohibitive ischemic risk of intervention, conservative management was selected.

Over the subsequent ICU course, the patient’s neurological examination gradually improved following cerebrospinal fluid diversion and hemodynamic optimization. She was successfully weaned from mechanical ventilation without evidence of delayed neurological decline. Serial CT imaging demonstrated stable ventricular size and no recurrent hemorrhage. At the time of discharge to inpatient rehabilitation, she was awake, following commands, and ambulating with assistance. Long-term angiographic follow-up was not available and is acknowledged as a limitation of this report.

## Discussion

ASA aneurysms represent one of the rarest subsets of intracranial aneurysms [4,6]. Most reported cases arise at the vertebral-ASA junction, a site predisposed to focal hemodynamic stress due to vessel angulation, pulsatile flow, and caliber mismatch between parent and branch vessels [3,9]. Age-related arterial wall degeneration and localized shear stress are thought to contribute to aneurysm formation even in the absence of inherited connective tissue disease [6].

CTA versus DSA

CTA remains the initial diagnostic modality of choice in suspected aneurysmal SAH because of its rapid availability and high sensitivity for common anterior circulation aneurysms [9]. However, CTA sensitivity decreases for aneurysms smaller than 3 mm and for lesions located in the posterior fossa [9,10]. In this case, CTA suggested the region of abnormality but could not definitively characterize the lesion. DSA provided definitive visualization of aneurysm morphology, parent-vessel relationships, and functional collateral supply, directly impacting management decisions [10].

Importance of ASA collateral assessment

The ASA is the dominant longitudinal arterial supply to the anterior spinal cord [5]. Inadvertent occlusion during endovascular treatment may result in anterior spinal artery syndrome with irreversible motor deficits [8]. In this patient, bilateral ASA supply was demonstrated angiographically; however, because the aneurysm arose directly from the ASA trunk itself, even partial compromise remained high risk. This anatomical configuration strongly favored conservative management.

Management strategy in context

While endovascular treatment of select ASA aneurysms has been reported [8,12], conservative management remains appropriate when ischemic risk exceeds hemorrhagic risk [13]. In this case, stabilization without rebleeding supports the selected management strategy, though definitive conclusions regarding long-term aneurysm stability cannot be drawn without follow-up angiography.

Limitations

This report is limited by the absence of long-term angiographic surveillance, the inherent measurement variability of sub-2 mm aneurysms [10], and the single-patient design. Continuous intracranial pressure trends, detailed laboratory data, and long-term functional outcome measures were not uniformly available for structured analysis.

## Conclusions

This report demonstrates that small ruptured vertebral-ASA junction aneurysms may evade definitive detection on CTA due to posterior fossa artifact and vessel overlap. DSA remains essential for accurate diagnosis, morphological characterization, and functional assessment of the ASA collateral supply. When aneurysms arise directly from the ASA trunk, the ischemic risk of endovascular intervention may outweigh the benefit of immediate occlusion. In such cases, individualized management guided by detailed angiographic anatomy is critical to minimizing catastrophic spinal cord injury.

## References

[1] Ziu E, Khan SMZ, Mesfin FB (2025). Subarachnoid Hemorrhage. https://www.ncbi.nlm.nih.gov/books/NBK441958/.

[2] Faluk M, Das JM, De Jesus O (2025). Saccular Aneurysm. StatPearls [Internet.

[3] Raz E, Shapiro M, Nossek E (2025). Neuroanatomy of the vertebrobasilar perforators: implications for aneurysm treatment. J Neurointerv Surg.

[4] Qu Y, Yu J (2025). Current status and prospects of endovascular treatment for intracranial vertebral artery aneurysms: a narrative review. Medicine (Baltimore).

[5] Ali F, Reddy V, Dublin AB (2025). Anatomy, Back, Anterior Spinal Artery. https://www.ncbi.nlm.nih.gov/books/NBK532963/.

[6] Yang TK (2013). A ruptured aneurysm in the branch of the anterior spinal artery. J Cerebrovasc Endovasc Neurosurg.

[7] Aboul-Ela HM, Salah El-Din AM, Zaater AA, Shehab M, El Shahawy OA (2018). Predictors of shunt-dependent hydrocephalus following aneurysmal subarachnoid hemorrhage: a pilot study in a single Egyptian institute. Egypt J Neurol Psychiatr Neurosurg.

[8] Larson AS, Mehta T, Grande AW (2021). Neurosurgical management of aneurysms of the vertebrobasilar system: increasing indications for endovascular therapy with a continued role for open microneurosurgery. Neurosurg Rev.

[9] Yang ZL, Ni QQ, Schoepf UJ (2017). Small intracranial aneurysms: diagnostic accuracy of CT angiography. Radiology.

[10] Vella M, Alexander MD, Mabray MC (2020). Comparison of MRI, MRA, and DSA for detection of cerebral arteriovenous malformations in hereditary hemorrhagic telangiectasia. AJNR Am J Neuroradiol.

[11] Frontera JA, Claassen J, Schmidt JM (2006). Prediction of symptomatic vasospasm after subarachnoid hemorrhage: the modified Fisher scale. Neurosurgery.

[12] Yasargil MG (1996). Microneurosurgery, Vol IV. Microneurosurgery, Vol IV: AVMs and.

[13] Zhao L, Zhao SQ, Tang XP (2021). Ruptured intracranial aneurysm presenting as cerebral circulation insufficiency: a case report. World J Clin Cases.

